# Nootkatone Inhibits Acute and Chronic Inflammatory Responses in Mice

**DOI:** 10.3390/molecules25092181

**Published:** 2020-05-07

**Authors:** Lindaiane Bezerra Rodrigues Dantas, Ana Letícia Moreira Silva, Cícero Pedro da Silva Júnior, Isabel Sousa Alcântara, Maria Rayane Correia de Oliveira, Anita Oliveira Brito Pereira Bezerra Martins, Jaime Ribeiro-Filho, Henrique Douglas Melo Coutinho, Fabíolla Rocha Santos Passos, Lucindo José Quintans-Junior, Irwin Rose Alencar de Menezes, Raffaele Pezzani, Sara Vitalini

**Affiliations:** 1 Departamento de Saúde, Centro Universitário Dr. Leão Sampaio-UNILEÃO, Av. Leão Sampaio, 400-Lagoa Seca, Juazeiro do Norte 63040-000, Ceará, Brazil; lindaianebrd@gmail.com (L.B.R.D.); leticia-drt@hotmail.com (A.L.M.S.); 2 Laboratory of Pharmacology and Molecular Chemistry, Department of Biological Chemistry, Regional University of Cariri, Rua Coronel Antônio Luis 1161, Pimenta, Crato 63105-000, Ceará, Brazil; juninhocatolico@hotmail.com (C.P.d.S.J.); isabel-alcantara-@hotmail.com (I.S.A.); rayaneoliveirabio@gmail.com (M.R.C.d.O.); anitaoliveira24@yahoo.com.br (A.O.B.P.B.M.); irwin.alencar@urca.br (I.R.A.d.M.); 3 Gonçalo Moniz Institute, Oswaldo Cruz Foundation, Salvador 45500-000, Bahia, Brazil; jaimeribeirofilho@gmail.com; 4 Microbiology and Biology Molecular Laboratory, Department of Chemical Biology, Regional University of Cariri, Crato 63105-000, Ceara, Brazil; hdmcoutinho@gmail.com; 5 Graduate Program in Health Sciences, Federal University of Sergipe, Aracaju, Sergipe 49100-000, Brazil; fabiollarsp@hotmail.com (F.R.S.P.); lucindojr@gmail.com (L.J.Q.-J.); 6 O.U. Endocrinology, Department of Medicine (DIMED), University of Padova, via Ospedale 105, 35128 Padova, Italy; raffaele.pezzani@gmail.com; 7 AIROB, Associazione Italiana per la Ricerca Oncologica di Base, 35128 Padova, Italy; 8 Department of Agricultural and Environmental Sciences, Milan State University, via G. Celoria 2, 20133 Milan, Italy

**Keywords:** nootkatone, anti-inflammatory, acute inflammation, granuloma

## Abstract

Nootkatone (NTK) is a sesquiterpenoid found in essential oils of many species of *Citrus* (Rutaceae). Considering previous reports demonstrating that NTK inhibited inflammatory signaling pathways, this study aimed to investigate the effects of this compound in mice models of acute and chronic inflammation. Murine models of paw edema induced by carrageenan, dextran, histamine, and arachidonic acid, as well as carrageenan-induced peritonitis and pleurisy, were used to evaluate the effects of NTK on acute inflammation. A murine model of granuloma induced by cotton pellets was used to access the impact of NTK treatment on chronic inflammation. In the acute inflammation models, NTK demonstrated antiedematogenic effects and inhibited leukocyte recruitment, which was associated with decreased vascular permeability, inhibition of myeloperoxidase (MPO), interleukin (IL)1-β, and tumor necrosis factor (TNF)-α production. In silico analysis suggest that NTZ anti-inflammatory effects may also occur due to inhibition of cyclooxygenase (COX)-2 activity and antagonism of the histamine receptor type 1 (H_1_). These mechanisms might have contributed to the reduction of granuloma weight and protein concentration in the homogenates, observed in the chronic inflammation model. In conclusion, NTK exerted anti-inflammatory effects that are associated with inhibition of IL1-β and TNF-α production, possibly due to inhibition of COX-2 activity and antagonism of the H1 receptor. However, further studies are required to characterize the effects of this compound on chronic inflammation.

## 1. Introduction

Inflammation is the body’s response against tissue damage triggered by a variety of endogenous and exogenous stimuli. Its purpose is to remove the harmful agent and restore tissue integrity. The inflammatory reaction involves a series of tissue changes, such as vasodilation with increased blood flow, increased vascular permeability with plasma exudation and protein extravasation, and recruitment of leukocytes in response to cytokines and chemokines. In this context, neutrophils and macrophages, which play critical roles in the early phase of inflammation, also contribute to the development of many chronic inflammatory diseases [1,2].

Inflammatory mediators include a great diversity of lipids (e.g., eicosanoids), proteins (e.g., cytokines, chemokines, and adhesion molecules) and other chemically related substances that orchestrate various cellular and tissue changes through binding to receptors on leukocytes, endothelial cells, and many other cell types [3]. Some of these mediators are preformed and stored in cytoplasmic granules of mast cells, basophils, and platelets while others circulate as inactive precursors in the plasma. However, most of them are produced directly in response to inflammatory stimuli that may cause severe tissue damage related to the activation of autolytic pathways [4,5]. Persistence of the stimulus, as well as continuous production of inflammatory mediators, cause significant tissue damage, which is associated with morphological and functional changes observed in the pathogenesis of several chronic diseases [6].

Cytokines are notable chemical mediators associated with the development of uncountable inflammatory events. These molecules are produced from gene transcription in immune and non-immune cells, exerting crucial roles in the host defense against pathogens; however, especially in response to severe tissue damage, intense production of cytokines can be potentially harmful [7].

In this context, the tumor necrosis factor (TNF)-α and interleukin (IL) and IL-1 have remarkable local and systemic effects. These cytokines are capable of activating leukocytes and other cell types, stimulating the production of chemical mediators that amplify the inflammatory response [7,8].

**Figure 1:**
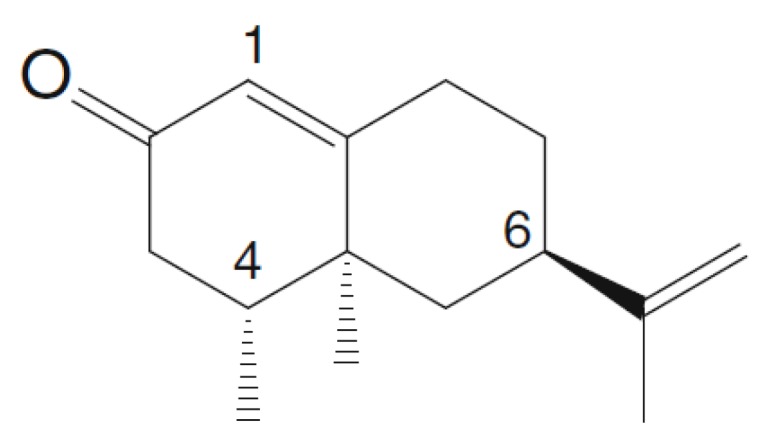
(+)-Nootkatone (NTK) [8].

Anti-inflammatory compounds, either synthetic or naturally occurring, are substances capable of inhibiting inflammatory events by interfering with signaling cascades associated with the production or action of inflammatory mediators. In most cases, their mechanism of action involves inhibition of enzymes (such as cyclooxygenase (Cox) -2 and 5- lipoxygenase (LO)), receptors or gene transcription. Corticosteroids and Non-Steroidal Anti-inflammatory Drugs (NSAIDs) are the main drug classes used to treat inflammatory symptoms. Nevertheless, they cause severe side effects, indicating the importance of developing novel, safe, and effective anti-inflammatory drugs [6,9].

(+)–Nootkatone (NTK) is a ketone terpenoid belonging to the largest subclass of sesquiterpenes (Figure 1) [10]. This naturally occurring organic compound is found as a component of aromatic species such as Alaskan cedar (*Cupressus nootkatensis* D.Don) and Java (*Cyperus rotundus* L.), as well as in essential oils of *Citrus* (Rutaceae), *Alpinia* (Zingiberaceae), *Chrysopogon* (Poaceae) and many other genera [11]. Due to its pleasant fragrance, this compound, as well as its biogenetic precursor valencene, have been industrially used in the chemical, food, and cosmetic sectors [11,12]. Nevertheless, studies have demonstrated that other components of citrus fruits, such as flavonoids, mainly due to their antioxidant and anti-inflammatory activities, contribute to the relevance of *Citrus* species as sources of natural products with both industrial and medicinal use. Additionally, as flavonoids are abundant in fruit juices, the consumption of these products represents an easy way to take advantage of the therapeutic properties of these compounds [12].

Previous studies demonstrated that plant extracts containing NTK presented anti-inflammatory effects. Tsoyi and collaborators demonstrated that NTK and valencene inhibited nitric oxide (NO) production by inhibiting NO synthase [13]. It was also found that the compound inhibited platelet activation [14] and lipid peroxidation, in addition to inhibiting systemic oxidative stress, as well as preventing DNA damage by activating nuclear factor erythroid-derived 2-like 2 and heme oxygenase 1 [15].

Considering the pharmacological properties of NTK, this study aimed to investigate its effects in mice models of acute and chronic inflammation. 

## 2. Results

### 2.1. NTK Inhibits Paw Edema Induced by Different Inflammatory Agents

**Figure 2:**
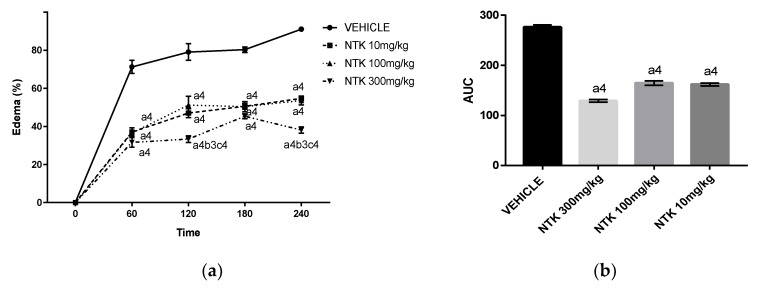

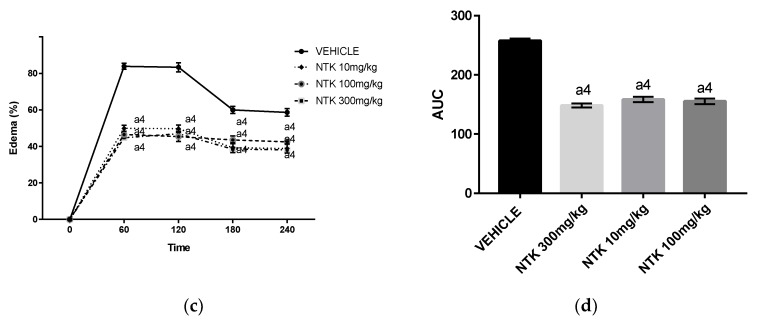
Time-point analysis of treatment with different NTK doses on paw edema formation induced by carrageenan (**a**) or dextran (**c**). These data are also represented as area under the curve (AUC) ((**b**,**d**), respectively)). a4 = *p* < 0.0001 vs. saline; b3 = *p* < 0.001 vs. NTK 100 mg/kg group; c4 = *p* < 0.0001 vs. NTK 10 mg/kg group. Statistical significance was determined with two-way ANOVA (**a**,**c**) and post hoc Tukey test.

Because edema is one of the first inflammatory cardinal signs in acute responses, we investigated the effects of NTK on paw edema induced by different triggering agents. Initially, we analyzed the effect of a single oral pre-treatment with varying doses of NTK (10, 100, and 300 mg/kg) at different time points after challenge with carrageenan (Figure 2a) or dextran (Figure 2c). These treatments caused significant inhibition of the edema caused by both agents at all evaluated time-points. In addition, as demonstrated by the analysis of the area under the curve (AUC) (Figure 2b,d), no significant difference among the doses was observed. Therefore, the lower dose (10 mg/kg) was used throughout the experiments.

**Figure 3:**
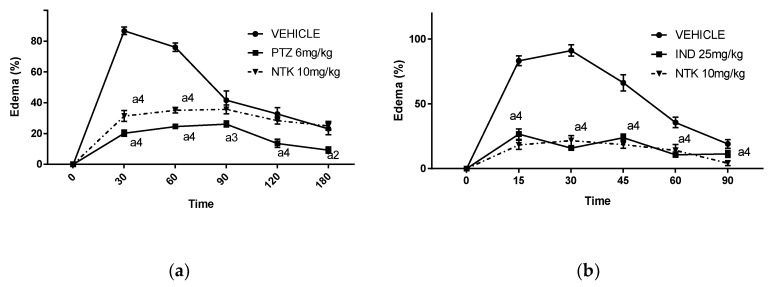
Potential mechanisms associated with NTK-mediated paw edema inhibition. The antiedematogenic effect is expressed as a percentage of edema induced by histamine (**a**) or arachidonic acid (**b**). a4 = *p* < 0.0001 vs. saline; a3 = *p* < 0.001 vs. saline and a2 = *p* < 0.01 vs. saline. Statistical significance was determined with two-way ANOVA and post hoc Tukey test.

Histamine and arachidonic acid (AA)-derived lipid mediators are crucially involved in edema formation during inflammatory conditions [16]. Therefore, we investigated the participation of these mediators on NKT-mediated edema inhibition. As shown in Figure 2a, a single oral pre-treatment with NTK (10 mg/kg) inhibited paw edema formation at 30 and 60 min after challenge in comparison with vehicle pre-treatment. Promethazine (PTZ) (6 mg/kg), a histamine receptor antagonist, caused similar inhibition of the edema, suggesting that NTK-mediated antiedematogenic effects may involve, at least partially, inhibition of histamine vascular actions. Accordingly, the most significant antiedematogenic effect of NTK was observed in AA-stimulated mice (Figure 3b). Moreover, indomethacin (IND), an NSAID used as a positive control, caused comparable inhibition at all evaluated time-points, suggesting that the antiedematogenic effects of NTK might involve arachidonic acid metabolism inhibition.

### 2.2. The Effects of NTK on Carrageenan-Induced Peritonitis

**Figure 4:**
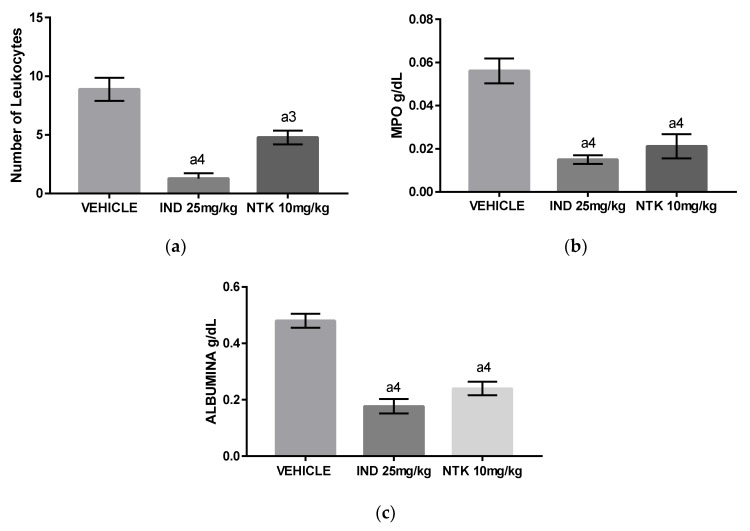
The effects of NTK on carrageenan-induced peritonitis. (**a**) The number of total leukocytes; (**b**) concentrations of myeloperoxidase (MPO), and (**c**) concentrations of albumin in the peritoneal fluid of mice. a4 = *p* < 0.0001 vs. saline and a3 = *p* < 0.001 vs. saline. Statistical significance was determined with one-way ANOVA and post hoc Tukey test.

The intraperitoneal administration of carrageenan (1%) induced an intense influx of leukocytes associated with elevated myeloperoxidase (MPO) and albumin levels into the peritoneal cavity of mice 4 h after challenge (Figure 4a–c), indicating the development a peritoneal inflammation associated with leukocyte migration and activation, and increased vascular permeability. The animals treated with NTK (10 mg/kg) or indomethacin (25 mg/kg) presented a significant reduction in all these inflammatory parameters, demonstrating that the treatments exerted anti-inflammatory effects in mice.

### 2.3. NTK Inhibits Leukocyte Recruitment and Cytokine Production on Carrageenan-Induced Pleurisy

**Figure 5:**
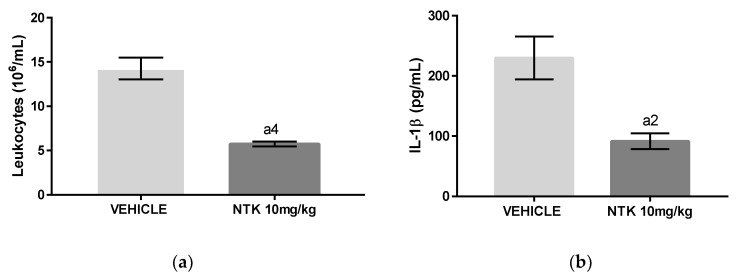

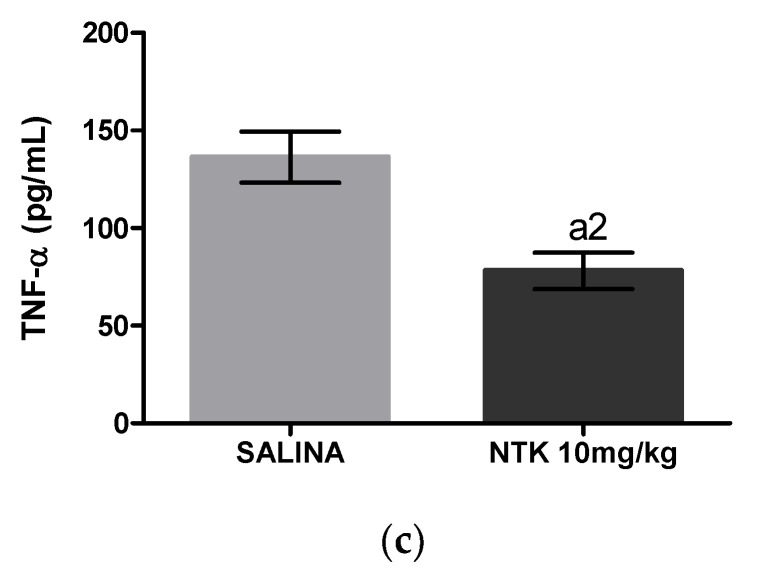
The effects of NTK on carrageenan-induced pleurisy. (**a**) The number of total leukocytes; (**b**) concentrations of Interleukin (IL)-1β, and (**c**) concentrations of Tumor Necrosis Factor (TNF)-α in the pleural lavages of mice. a4 = *p* < 0.0001 vs. saline and a2 = *p* < 0.01 vs. saline. Statistical significance was determined with one-way ANOVA and post hoc Tukey test.

Interleukin (IL)-1β and Tumor Necrosis Factor (TNF)-α have critical roles in the development of acute inflammation [17]. The group of mice challenged with carrageenan and pre-treated with NTK (10 mg/kg) presented a significantly reduced number of total leukocytes associated with lower concentrations of IL1-β and TNF-α in the pleural lavages in comparison with untreated animals (Figure 5a–c), indicating that inhibition of cytokine production is potentially related with NTK anti-inflammatory mechanisms.

### 2.4. Investigation of NTK-Mediated COX-2 and H_1_ Inhibition in Silico

In the present study, the docking procedure was validated by removing and repositioning each ligand into the binding site. The root-mean-square deviation (RMSD) resulting from the X-ray crystallography structures found conformations of 0.87Å and 0.32Å (for COX-2 and histamine, respectively), demonstrating convergence in the calculated complex formation and attesting the performance of the docking protocol. The results of docking analysis determine how closely the lowest energy pose (binding conformation) from the COX-2 enzyme and the H_1_ receptor. 

**Figure 6:**
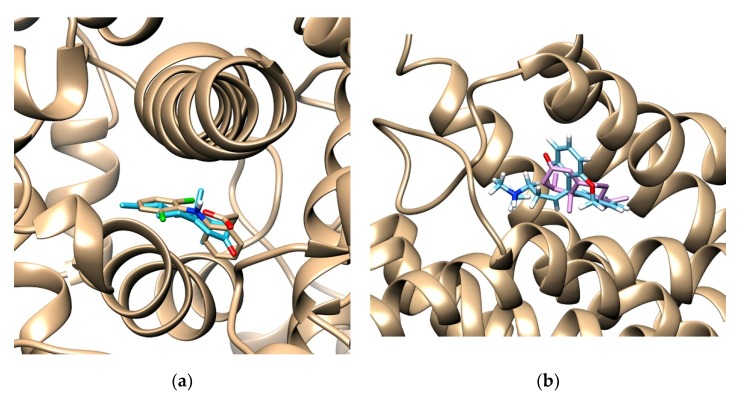
The binding poses of best stability between NTK and diclofenac into the cyclooxygenase (COX)-2 enzyme binding site (**a**) and between NTK and doxepin into the binding site of the H_1_ receptor (**b**).

**Figure 7:**
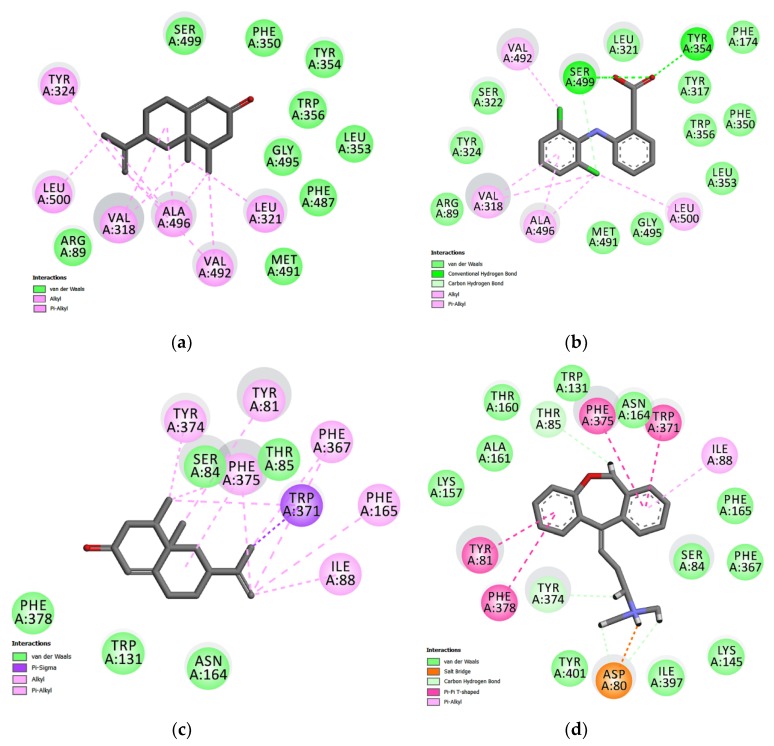
Maps of amino acid residues within the binding pocket of COX-2 (**a**,**b**) and the H1 receptor (**c**,**d**). The interactions of NKT (**a**,**c**), Diclofenac (**b**), and Doxepin (**d**) with these amino acids is illustrated.

The docked ligands showed score energies of −8.6 kcal/mol to diclofenac and −8.0 kcal/mol for nootkatone in the COX-2 binding site. Considering the H_1_ receptor, the score energies were −11.5 kcal/mol for doxepin and −8.1 kcal/mol for nootkatone. Of note, these data were useful to analyze the hydrophobic and polar interactions in the binding site of the complexes. The best conformation of nootkatone into COX-2 enzyme and H_1_ receptor binding sites indicate a favorable interaction with both proteins (Figure 6a,b), as well as their respective control ligands diclofenac and doxepin, suggesting a link between COX-2 and H_1_ inhibition and the NTK-mediated anti-inflammatory effects (Figure 7a–d).

### 2.5. The Effects of NTK on Cotton Pellet-Induced Granuloma

**Figure 8:**
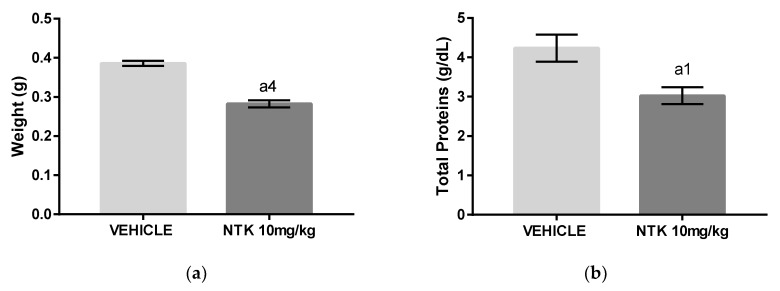
The effects of NTK treatment on cotton pellet-induced granuloma in mice. (**a**) Final weight of the granuloma. (**b**) Protein concentration in the homogenates. a4 = *p* < 0.0001 vs. saline and a1 = *p* < 0.05 vs. saline. Statistical significance was determined with T test.

The cotton pellet-induced granuloma model was used to evaluate the effects of NTK on chronic inflammation. Administration of NTK (10 mg/kg) to mice decreased both granuloma weight (Figure 8a) and concentration of proteins in the homogenates (Figure 8b) in comparison with untreated mice, indicating that the treatment inhibited, at least partially, the inflammatory response in this model.

## 3. Discussion

The present study demonstrated the anti-inflammatory effects of NTK, an aromatic sesquiterpenoid widely found in the plant kingdom. A screening evaluating the effect of different doses of this compound on paw edema induced by carrageenan or dextran, demonstrated significant antiedematogenic effects at 10, 100, or 300 mg/kg in mice. Therefore, we chose the lower dose for the following analysis. Carrageenan and dextran are phlogistic agents that act by triggering an inflammatory reaction characterized by the release of chemical mediators, such as prostaglandins (e.g., PGE_2_) and vasoactive amines (e.g., histamine), which increase the vascular permeability and cause the biochemical, cellular, and vascular changes observed in acute inflammation [18]. 

Considering the involvement of vasoactive amines and lipid mediators on edema induction, we investigated the effects of NTK pre-treatment on edema triggered by histamine and AA challenge. It was shown that the compound partially inhibited paw edema formation triggered by histamine administration and promethazine, a histamine receptor antagonist, caused comparable inhibition, suggesting that NTK-mediated antiedematogenic effects may involve, at least partially, inhibition of histamine vascular actions. NTK Pre-treatment significantly inhibited edema formation in AA-stimulated mice. Accordingly, indomethacin, an NSAID used as the positive control, caused comparable inhibition at all evaluated time-points, suggesting that the effects of NTK in acute inflammation might involve arachidonic acid metabolism inhibition, possibly through interference with the enzymatic activity of cyclooxygenase (COX), lipoxygenase (LO), or phospholipase A_2_ (PLA_2_), or even by antagonizing the effects of bioactive eicosanoids on tissue receptors. Nevertheless, the similarity in magnitude between the effect of NTK and indomethacin, suggests a common action on the COX pathway [19,20]. Our findings are corroborated by previous research demonstrating that NTK concentration-dependently inhibited platelet activation induced by AA in vitro. Since prostaglandins significantly mediate AA-induced platelet activation, we hypothesized that NTK could be acting through inhibition of COX activation in platelets [14].

To investigate the involvement of COX-2 and H_1_ receptor inhibition on NTZ-mediated anti-inflammatory effects, we performed in silico analysis by molecular docking. The COX-2 binding site shows two important regions of interaction with inhibitors: the hydrophobic pocket formed by Tyr324, Trp356, Phe487, Phe350, Ala496, Tyr317, and Leu321, and the hydrogen bond pocket formed by Ser-499 and Tyr-354. The Alkyl and Alkyl-pi stacking interactions work as “anchors”, favoring the van der Waals interactions and contributing to the formation of a stable bond in the COX-2/NTK complex. Our results demonstrated a complementarity between the ligands and the active site of COX-2. The relative contribution of van der Waals interactions has demonstrated to be relevant for both NTK and diclofenac. In addition, 15 similar interaction points with hydrophobic and hydrophilic cavities were found between NTK and diclofenac. However, the control drug had relatively better docking energy, in part, generated by hydrogen bonds. Previous studies with other terpenes derivatives have demonstrated that these compounds interacted similarly, although no involvement of hydrogen bonds was shown [21,22]. In the H_1_ receptor binding site, NTK showed potential Van der Waals interactions with Ser84, Thr 85, Phe 378, Trp 131, Asn 164 (Figure 7). However, other interactions such as alkyl, π-sigma, and π-alkyl, contribute to the stabilization of the interaction, as well as to the formation of the lipophilic pocket in the binding site. According to Keserű, G. M. et al. (2004), taking part in the lipophilic pocket formation is crucial for the antagonist activity in the binding cavity. The interactive map shows nine similar stabilization points between the NTK/H_1_ and the doxepin/H_1_ complex at the binding site. Therefore, NTK shows favorable docking with the COX-2 enzyme and the H_1_ receptor. These amino acid similarities support the hypothesis that NTK occupies the active site in both targets, corroborating our experimental data using in vivo models.

Carrageenan-induced pleurisy and peritonitis are well-established models used for evaluation of the systemic anti-inflammatory properties of natural and synthetic drugs. A single oral pre-treatment with NTK (10 mg/kg) caused significant inhibition of leukocyte recruitment associated with decreased concentrations of albumin, MPO, IL1-β, and TNF-α, demonstrating that the compound inhibited hallmark parameters of acute systemic inflammation. The decreased albumin levels found in NTK-treated animals indicates an inhibitory action by this compound on carrageenan-mediated increased vascular permeability. Accordingly, the lower concentrations of IL-1β and TNF-α found in the NTK group justify, at least partially, the inhibitory effects of this compound on all acute inflammation parameters demonstrated by this study, since these cytokines were shown to exert multiple inflammatory actions, leading to increased vascular permeability and leukocyte recruitment and activation [22]. In this context, in addition to presenting a reduced number of leukocytes in both pleural and peritoneal lavages, the NTK-treated mice showed significantly reduced MPO levels, indicating inhibition of leukocyte activity. 

In order to evaluate the effects of NTK against a chronic stimulus, we used the cotton pellet-induced granuloma model in mice. NTK treatment for 10 consecutive days showed positive effects, reducing both granuloma weight and total proteins in the homogenates. These findings suggest a possible inhibitory action of the treatment on macrophage migration and activation as well as of the proliferative response, corroborating the effects of NTK on cytokine production and leukocyte activity. Importantly, because NTK was found to reduce several inflammatory parameters induced by different noxious stimuli in different mice models of acute systemic inflammation, we suggest that this sesquiterpene may be used for further research aimed at the development of new anti-inflammatory drugs. 

The results obtained in the present research are corroborated by the findings of previous studies. Choi et al. [20] demonstrated in vitro that NTK suppressed the expression of chemokines such as thymus and activation-regulated chemokine (TARC/CCL17) and macrophage-derived chemokine (MDC/CCL22) in HaCaT cells, by inhibiting the mitogen-activated protein kinases (MAP kinases) PKC and p38. Since these kinases participate in signaling pathways involved in the activation of the transcriptional nuclear factor-kappaB (NF-κB) transcriptional factor, these findings suggest that NKT could modulate the inflammatory response at multiple levels, since NF-κB stimulates the transcription of numerous genes from cytokines, chemokines and adhesion molecules, supporting the findings of the present study. According to Qi and collaborators [23], the NKT treatment reduced the levels of TNF-α, IL-1β, and IL-6 in models of cognitive impairment and dementia in rats, corroborating the data from our pleurisy model. Moreover, NKT significantly reduced the activities of inflammatory enzymes such as superoxide dismutase (SOD), glutathione S-transferase (GST), cyclooxygenase-2 (COX-2), and inducible nitric oxide synthase (iNOS) and levels of glutathione (GSH), in addition to decreasing the total antioxidant capacity (T-AOC), as well as concentrations of malondialdehyde (MDA) and nitric oxide (NO) through inhibition of the Toll-like receptor 4/NF-κB/domains-containing-protein-3-inflammasome (TLR4/NF-κB/NLRP3) pathway [23]. NTK (90 mg Kg) was also shown to reduce the anti-inflammatory effects of cigarette vapor [24], as well as inhibit lung inflammation parameters via NF-κB inhibition [19]. Nevertheless, the mechanisms underlying the effects of NTK as an anti-inflammatory compound remain to be fully characterized. Therefore, we will carry out in vitro studies to analyze the impact of NTK on COX-1 and COX-2 expression and activity, which will be correlated with the levels of PGE_2_ in the supernatants of macrophage cultures. Additionally, we will use mast cell cultures will to analyze the effects of NTK on histamine release in vitro, which will be correlated with the activity of this compound in combination with agonists and antagonists of the H_1_ receptor in vivo.

Finally, Kurdi and colleagues demonstrated that NTK (10 mg/kg) treatment exerted hepatoprotective and antifibrotic effects by suppressing the carbon tetrachloride (CCL4)-induced lesion, which is characterized by expression of pro-inflammatory cytokines, such as TNF-α, monocyte chemotactic protein (MCP)-1 and IL-1β in liver tissues. In the same study, histopathological findings revealed that NTK reduced fibrosis, steatosis, hepatocyte necrosis and leukocyte infiltration [25], providing evidence that NTK could be useful in the management of chronic inflammation, as indicated by our findings in the cotton pellet-induced granuloma. However, further research is required to characterize better the anti-inflammatory effects of NTK, as well as its mechanism of action on chronic inflammation. To this end, we will carry out experiments to characterize the cell populations, as well as the expression of chronic inflammation markers in the granuloma model. 

In conclusion, NTK presented anti-inflammatory effects in mice models of acute and chronic inflammation. In acute inflammation, the antiedematogenic effects, as well as leukocyte recruitment inhibition, may be associated with decreased vascular permeability and inhibition of MPO, IL1-β, and TNF-α production, possibly due to inhibition COX-2 activity and antagonism of the H1 receptor. These mechanisms might have contributed to the anti-inflammatory effects displayed by NTK on the granuloma model. However, further studies are required to characterize the effects of this compound on chronic inflammation.

## 4. Materials and Methods

### 4.1. Drugs and Reagents

Compound NTK, all inflammatory agents (carrageenan, dextran, histamine, and arachidonic acid), ELISA kits from eBioscience and compound o-dianisidine (used for myeloperoxidase assay) were purchased from Sigma-Aldrich (New York, NY, USA); the kits for albumin and total proteins quantification were supplied by Labtest (Lagoa Santa, Brazil), ketamine was purchased by the VETNIL (São Paulo, Brazil), and xylazine was purchased by CEVA (São Paulo, Brazil).

### 4.2. Animals

Swiss mice (Mus musculus) of both sexes, weighing 20–30 g, were randomly assigned into groups and maintained in polypropylene cages at 22 ± 3 °C, under a 12 h light/dark cycle and with free access to water and food (Labina, Purina^®^, Euskirchen, Germany). The research was carried out in accordance with the guidelines animal testing (Guide for the care and use of laboratory animals, from NIH—National Institute of Health—USA, 1996; Federal Law No. 11,794/2008 and National Experiment Control Council—CONCEA). The protocols used in this study were approved by the local institutional review board for animal experimentation (CEUA, Regional University of Cariri, protocol number 100/2019.2).

### 4.3. Evaluation of the Anti-Inflammatory Activity

The effects of NTK on acute inflammation were evaluated using the following mice models: carrageenan-induced peritonitis, carrageenan-induced pleurisy, and paw edema induced by either carrageenan, dextran, histamine, or arachidonic acid (AA). In all these protocols, the mice received a single oral treatment one hour before the challenge. Each challenge was performed through a single administration of an inflammatory agent (carrageenan, histamine, dextran, or AA) to the mice, triggering an acute inflammatory response, which was monitored immediately after the challenge at specific time-points, according to standard protocols. The paw edema models were used to evaluate the local inflammatory response, while pleurisy and peritonitis were used to analyze systemic inflammatory parameters. In the chronic inflammation protocol, the animals were treated daily for ten consecutive days, after surgically receiving a persistent stimulus (cotton pellets). This protocol induces the formation of granuloma, which is considered as a parameter of chronic inflammation.

#### 4.3.1. Evaluation of the Antiedematogenic Activity

The animals (*n* = 6 per group) were randomly assigned into groups and orally treated with NTK (10, 100, or 300 mg/kg), vehicle (0.9% saline, negative control) or control drugs (promethazine, 10 mg/kg or indomethacin, 25 mg/kg) 1 h before challenge with 20 µL of carrageenan, dextran, histamine, or AA at 1% (*w*/*v*). Each inflammatory agent used in the challenge was administered in the hind right paw and an equal volume of saline was administered in the hind left paw. The volume of both paws was measured at different time-points according to the type of challenge, using a plethysmometer and the results were expressed as a percentage of edema, considering the difference between the hind and left paw of the untreated group as 100% [19,21,22].

#### 4.3.2. Carrageenan-Induced Peritonitis

The mice (*n* = 6) were orally pre-treated with the vehicle, NTK (10 mg/kg), or indomethacin (25 mg/kg) 1 h before receiving an intraperitoneal injection of 1 mL of 1% carrageenan (challenge). Four hours after challenge, these animals were euthanized by CO_2_ inhalation, the peritoneal cavity was washed with 3 mL of heparinized PBS (10 IU/mL), and the peritoneal lavage was collected for leukocyte counting and quantification of MPO and total proteins [26]. Total leukocyte counts were performed using the SDH-20 model automatic counter and the differential counts were performed by optical microscopy, on slides stained using the panoptic method.

#### 4.3.3. Carrageenan-Induced Pleurisy

The mice (*n* = 6) were orally pre-treated with the vehicle, NTK (10 mg/kg), or indomethacin (25 mg/kg) 1 h before receiving an intrathoracic injection of 1% carrageenan (challenge). Four hours later, the animals were anesthetized with ketamine (8 mg/kg) and xylazine (8 mg/kg) and euthanized by cervical dislocation. The pleural cavity was washed with 1 mL of saline containing 0.9% Ethylenediaminetetraacetic acid (EDTA, Sigma, New York, NY, USA). The pleural lavages were centrifuged (5000 rpm, 5 min, at room temperature), and supernatants were stored (at −80 °C) for further analysis. The precipitate was added with 1 mL of PBS, and leukocytes were counted under light microscopy after diluting the pleural lavage samples in Turk fluid (2% acetic acid) [27].

#### 4.3.4. Quantification of MPO and Total Proteins

Samples of the peritoneal lavage were centrifuged at 6000 RPM for 2 min. Albumin quantification in the supernatants was performed using a Labtest kit (Lagoa Santa, Brazil) and used as a parameter of protein extravasation due to increased vascular permeability. In this method, the absorbance of bromocresol green, which specifically binds albumin, is proportional to the concentration of the protein in the sample. The readings were performed using a spectrophotometer with absorbance adjusted between 600 and 640 nm. Leukocyte activity was determined by quantifying the MPO enzyme. To this end, a tube was added with 40 µL of the supernatant of the pleural lavage and 1960 µL of the o-dianisidine and H_2_O_2_ reagent in PBS (pH = 6.0). The readings were performed using a spectrophotometer at 450 nm.

#### 4.3.5. Cytokine Quantification

The concentrations of TNF-α and IL-1β were determined using enzyme-linked immunosorbent assay (ELISA) kits (Invitrogen^®^, California, CA, USA) according to the manufacturer’s instructions. Briefly, supernatants of pleural lavages were diluted 1:1 (*w*/*w*) in the diluent solution provided by the ELISA kit, and the readings were performed at 450 nm using a microplate reader (ASYS^®^, New York, NY, USA). The concentrations were obtained by interpolation from a standard curve and data were expressed as pg/mL.

#### 4.3.6. Cotton Pellet-Induced Granuloma

Mice (*n* = 6) previously anesthetized with ketamine (80 mg/kg) and xylazine (20 mg/kg) had four cotton pellets (0.01 g each) implanted through a small dorsal incision. Twenty-four hours later, the animals were treated orally with vehicle or NTK (10 mg/kg) for ten consecutive days. On day 11, the animals were euthanized, and the pellets, as well as the surrounding tissue, were removed, dried at 37 °C for 24 h, and weighed. The results were expressed as the difference between the final weight and the initial weight [28]. 

For total protein quantification, the pellets were placed in test tubes and homogenized with 1 mL of 0.9% saline. The concentration of total proteins was determined using a specific kit (Labtest, Lagoa Santa, Brazil) that is based on the reaction with copper ions in an alkaline medium, creating a violet color complex whose absorbance is proportional to the concentration of proteins in the sample. The readings were performed at 550 nm using a spectrophotometer.

### 4.4. In Silico Analysis of COX-2 Inhibition

Docking simulations were carried out for ligand-bound protein complexes, obtained from the Protein Data Bank (PDB, ID. 1PXX and 3RZE). The complexed structures were adjusted using a protein preparation tool provided by the Chimera package, the 3D structures of ligands were obtained using the corina^®^ 3D structure generator and minimization of energy was achieved using the UCSF Chimera structure build module. The binding region was defined by a 10 Å × 10 Å × 10 Å box set at the centroid of the co-crystallized ligand in the crystal complex to explore a large region of the enzyme structure. The docking analysis was carried out using the UCSF Chimera and AutoDock Vina software based on the Iterated local search global optimizer. Proteins and ligands were maintained flexible during the docking process. The selection of flexible residues from proteins was based on the active site at 4.0 Å from the co-crystallized ligands. The most favorable binding free energy was represented by clustering the positional RMSD results with not more than 1.0 Å. The final docked complexes were analyzed using Discovery Studio 3.1 visualizer [29].

### 4.5. Statistical Analyses

Data were analyzed by one-way ANOVA or two-way ANOVA and Tukey post hoc test or T test using GraphPad Prism software version 7.00 (GraphPad, San Diego, CA, USA, 2016). Values are expressed as means ± SEM. Statistical significance was considered when *p* < 0.05.
